# PlexProbes enhance qPCR multiplexing by discriminating multiple targets in each fluorescent channel

**DOI:** 10.1371/journal.pone.0263329

**Published:** 2022-03-09

**Authors:** Nicole Hasick, Ryung Rae Kim, Yin Xu, Simon Bone, Andrea Lawrence, Claire Gibbs, Nathan Danckert, Alison Todd

**Affiliations:** 1 SpeeDx Pty Ltd, Eveleigh, New South Wales, Australia; 2 Sydney Institute of Agriculture, School of Life and Environmental Sciences, Faculty of Science, The University of Sydney, Sydney, Australia; GGD Amsterdam, NETHERLANDS

## Abstract

The probe technology described in this paper facilitates detection and discrimination of multiple targets in a single fluorescent channel during PCR. This provides a strategy for doubling the number of targets that can be analysed simultaneously on existing PCR instruments. These probes are referred to as PlexProbes and produce fluorescence that can be switched ‘on’ or ‘off’ in the presence of target by manipulating the temperature. During PCR, fluorescence can be measured at multiple temperatures allowing discrimination of specific targets at defined temperatures. In a single fluorescent channel, a model duplex assay allowed either real-time or endpoint detection of *Chlamydia trachomatis* (CT) at 52°C and end-point detection of *Neisseria gonorrhoeae* (GC) at 74°C. Using this model system, as few as 40 copies of each specific target could be detected as single infection or co-infection, regardless of the presence or absence of the other target. A PlexProbe prototype assay for sexually transmitted infections (PP-STI) which simultaneously enables detection and differentiation of six targets using only three fluorescent channels was then constructed and evaluated. The PP-STI assay detects GC (2 gene targets), CT, *Mycoplasma genitalium* (MG), *Trichomonas vaginalis* (TV) and an internal control (IC). To evaluate assay performance, a panel of archived clinical samples (n = 337) were analysed using PP-STI and results compared to those obtained with a commercially available diagnostic assay. The overall agreement between results obtained with the PP-STI assay and the reference test was greater than 99.5%. PlexProbes offer a method of detecting more targets from a single diagnostic test, empowering physicians to make evidence-based treatment decisions while conserving time, labour, sample volume and reagent costs.

## Introduction

Since its advent, PCR continues to be a popular method across a wide breadth of scientific fields with a myriad of applications [1, 2]. In medical diagnostics, the ability to amplify and detect multiple targets in a single PCR reaction is no longer a goal, but an expectation as it facilitates faster and more informative diagnosis of disease while also saving on time, labour, sample volume and reagent costs [3–5]. qPCR multiplexing can be achieved using a range of different probe-based technologies including Molecular Beacons, hydrolysis probes (e.g. TaqMan^®^), Scorpion probes, FRET probes, Catcher-Pitcher probes and PlexZymes (MNAzymes) [6–8].

PlexPCR^®^ is a qPCR method that employs target-dependant catalytic nucleic acids, known as PlexZymes^®^, to cleave universal PCR probes to generate fluorescence in the presence of target. During PlexPCR, primers amplify target nucleic acid sequences and produce amplicons which serve as a template for PlexZyme formation. Once the inactive PlexZyme components (known as Partzymes) have formed active PlexZyme enzymes, they can bind and cleave fluorescent-labelled universal probes to generate fluorescence [7, 8]. Changes in fluorescence allow detection and/or quantification of the target nucleic acid in real time. The assembly of multiple components (primers, Partzymes and probe) combined with the multiple turn-over capabilities of the enzyme provides 4-levels of specificity without compromise to assay sensitivity. Further, the universal nature of PlexPCR probes removes the need for target-specific probes, thereby making the development of new assays simpler and more affordable.

Currently, one of the main challenges for multiplexing, is that qPCR machines have a limited number of fluorescent channels, which typically range from one to six channels [9]. Furthermore, many point of care (POC) PCR instruments only have one or two fluorescent channels. Most PCR approaches, including PlexPCR, only permit identification of one target per fluorescent channel. Thus, the aim of the current study was to expand the multiplexing potential of PlexPCR to be able to differentiate multiple targets per detection channel.

To date, there are only a limited number of publications that demonstrate the ability to identify multiple targets in a single channel, with most of these using Melt Curve Analysis (MCA) to distinguish individual targets in a sample [10–16]. While this can increase the number of targets identified in each channel, MCA requires additional cycling time and is performed post-PCR, thus precluding the opportunity to quantify multiple targets in cases of co-infection. The method described in this study partially overcomes this limitation as it allows for the quantification of one target and qualitative determination of an additional target without the additional MCA cycling time.

This paper describes a new method for detecting and differentiating multiple targets in a single fluorescent channel using novel universal PlexProbes. PlexProbes are self-quenching fluorescent probes that can be used with PlexZymes [7, 8] to build highly sensitive and specific multiplex reactions. These probes are an extension of PlexPCR probes with the advantage of detecting and identifying multiple targets in each channel through their ability to reliably turn fluorescence ‘on’ or ‘off’ at distinct temperatures in response to target nucleic acids.

Sexually transmitted infections (STIs) are an increasing global burden with more than 1 million cases acquired every day worldwide [17]. *Chlamydia trachomatis* (CT), *Neisseria gonorrhoeae* (GC), *Mycoplasma genitalium* (MG) and *Trichomonas vaginalis* (TV) are four common pathogens that cause STIs. Due to the diversity of symptoms that are frequently non-specific, and since a substantial number of infections are asymptomatic, accurate diagnosis and prompt treatment are paramount for preventing complications such as pelvic inflammatory disease, cervicitis, sterility, and infertility [18–20]. Furthermore, accurate diagnosis and treatment of STIs are essential for controlling transmission and for reducing their burden within communities [19].

Here we demonstrate a novel strategy combining PlexProbes and standard linear PlexZyme probes [7] for detecting and differentiating two sexually transmitted bacteria per fluorescent channel without the need for MCA. We first demonstrate this using a model assay for detecting CT and GC in a single channel. Additionally, we assess the ability of a prototype PlexProbe assay (PP-STI) to detect a panel of key sexually transmitted pathogens (CT, GC, TV and MG) in clinical specimens by comparing its performance with that of a commercially available PCR kit.

## Materials & methods

### PlexProbe thermocycling protocol

PlexProbe PCR was performed on the LightCycler^®^ 480 Instrument II (Roche Diagnostics, Switzerland) using the following thermocycling protocol (Table 1).

**Table 1:** Thermocycling conditions for PlexProbe PCR.

Program Name	Cycles	Temperature (°C)	Hold Time	Ramp^≠^
Polymerase activation	1	95°C	2 min	4.4°C/s
Pre-PCR acquisition^+^	1	52°C^**+**^	15 sec	4.4°C/s
		74°C^**+**^	15 sec	
		85°C^**+**^	15 sec	
Touch down cycling: Step down -0.5°C/Cycle	10	95°C	5 sec	4.4°C/s
		61°C– 56.5°C	30 sec	2.2°C/s
Quantification cycling^+^: Acquisition/Detection	40	95°C	5 sec	4.4°C/s
		52°C^**+**^	40 sec	2.2°C/s
Post-PCR acquisition^+^	1	52°C^**+**^	3 min	4.4°C/s
		74°C^**+**^	15 sec	
Cooling	1	40°C	30 sec	2.2°C/s

^≠^ Ramp rate for 96 well plate

^+^ **Analysis mode:** Quantification, **Acquisition mode:** Single.

### Data analysis

The fluorescence at 52°C and quantification cycle (Cq) values for each well/sample were exported from the instrument analysis software and collated and analysed using MS Excel. Endpoint data at 52°C and 74°C were analysed using Formula A and B below and classified according to pre-determined threshold values (MG Positive >0.47, MG negative <0.47, TV Positive >1.9, TV negative <1.9, CT Positive >0.50, CT negative <0.5). Statistical agreement calculations were performed using the online diagnostic test evaluation and comparison calculator (Sergeant, ESG, 2018. Epitools Epidemiological Calculators. Ausvet. available at: http://epitools.ausvet.com.au.). Formula (A) and (B) where ERF is Endpoint Relative Fluorescence and F is fluorescence.


$$\left(A\right)ERF\;at\;52{}^{\circ}C=\frac{PostPCR\;F\;at\;52{}^{\circ}C-PrePCR\;F\;at\;52{}^{\circ}C}{PrePCR\;F\;at\;85{}^{\circ}C-PrePCR\;F\;at\;52{}^{\circ}C}$$

$$\left(B\right)ERF\;at\;74{}^{\circ}C=\frac{\left(PostPCR\;F\;at\;74{}^{\circ}C-PrePCR\;F\;at\;74{}^{\circ}C\right)}{\left(PrePCR\;F\;at\;85{}^{\circ}C-PrePCR\;F\;at\;52{}^{\circ}C\right)}-\frac{\left(PostPCR\;F\;at\;52{}^{\circ}C-PrePCR\;F\;at\;52{}^{\circ}C\right)}{\left(PrePCR\;F\;at\;85{}^{\circ}C-PrePCR\;F\;at\;52{}^{\circ}C\right)}$$

### Model assay detecting CT/GC in a single channel

PCR was performed in triplicate reactions which contained all components for amplification and detection of CT and GC in a total volume of 20 μL. Each reaction contained 8 mM MgCl_2_ (Bioline), 0.2 mM dNTPs (Bioline), 2 units MyTaq HS DNA polymerase (Bioline) and 1x NH_4_ Buffer (Bioline), 40 nM of each forward primer, 200 nM of each reverse primer, 200 nM of each partzyme A, 200 nM of each partzyme B and 200 nM of each probe. A linear PlexZyme probe is cleavable in the presence of CT whilst the PlexProbe is cleavable in the presence of GC. For this study, PlexProbe stems were designed so the intact probe has a Tm greater than 74°C and the cleaved probe has a Tm between 52°C and 74°C. The PlexProbe designs used in this study are universal and can be used without modification in panels detecting any nucleic acid target of choice. Additionally, all reactions contained either 10,000 or 40 copies of synthetic DNA template (G-Blocks^™^ gene fragments, Integrated DNA Technologies, Singapore) homologous to a portion of the target genes CTcry and/or GCopa, or nuclease-free water (NF H_2_O) representing the no template control. Reactions also contained a background of 10,000 copies of human genomic DNA (Promega) to mimic a clinical sample. The sequences of primers, partzymes, probes and targets for the CT/GC duplex assay are listed in S1 Table.

### Evaluation of PP-STI on clinical specimens

#### Clinical specimens

This study included a sample set of 341 DNA extracts selected from primary samples that had been submitted for routine clinical testing at the Vall d’Hebron University Hospital (HUVH) in Barcelona, Spain. Samples were selected for the evaluation study based on their clinical result of routine screening using the AllPlex^™^ STI essential assay (Seegene, South Korea), ensuring there was a variety of sample types and a sufficient number of positive and negative specimens for each pathogen. Samples were collected from male and female patients aged between 14 and 75. Four samples were excluded from the study due to inability to amplify the internal control (IC), therefore a total of 337 samples were statistically analysed. Sample types included 71 cervical swabs, 55 first void urine, 50 pharyngeal swabs, 50 rectal swabs, 40 urethral swabs and 71 vaginal swabs. Of the 337 specimens, 238 were positive for one pathogen, either CT (48), GC (77, TV (41) or MG (72). There were two specimens positive for both CT and TV and one specimen was positive for GC and CT. A total of 96 specimens were negative for all pathogens (Table 2).

**Table 2:** Results reported for specimens analysed using the AllPlex^™^ STI essential assay as part of routine screening.

Number of Specimens (n = 337)	Cervical swab (n = 71)	First void urine (n = 55)	Pharyngeal swab (n = 50)	Rectal swab (n = 50)	Urethral swab (n = 40)	Vaginal swab (n = 71)	Overall Results
CT	15	10	4	2	2	15	**48**
MG	19	15	0	15	3	20	**72**
GC	2	12	29	16	17	1	**77**
TV	18	2	0	1	1	19	**41**
TV and CT	1	0	0	0	0	1	**2**
CT and GC	0	1	0	0	0	0	**1**
Negative	16	15	17	16	17	15	**96**

Swab and urine samples were collected using the DeltaSwab ViCUM^®^ (Deltalab, Spain, ref: 304278) and in Jumbo Test Tube, Urine Collection tube (Vacutest KIMA, Italy, ref: 149640) respectively. There was no sample pre-treatment performed prior to nucleic acid extraction. Nucleic acid extraction and PCR set-up was performed on the STARlet IVD using the STARMag 96 Universal Cartridge kit (Seegene, South Korea). The sample input volume was 300 μL and the elution volume was 100 μL. DNA extracts were stored at -80°C for up to 2 years prior to being thawed and reanalysed using the PP-STI assay. Real-time PCR was performed in a CFX-96 real-time thermocycler (Bio-Rad, CA, USA), according to the manufacturer’s instructions (https://www.seegene.com/assays/allplex_sti_essential_assay). The Essential STI Allplex Assay is a multiplex qPCR assay for simultaneous detection of CT, GC, MG, TV, M. hominis (MH), U. urealyticum (UU), U. parvum (UP) and an IC and uses MuDT technology [21].

#### PP-STI assay

The PP-STI assay contained all the components for multiplexed amplification and detection of CT, GC (two genes), MG, TV and an IC. The assay was performed in 96 well plates using a 20 μL final volume with 15 μL of master mix, which included IC DNA template (40 copies), and 5 μL of extracted sample. The single well assay detects six targets using three fluorescent channels. The target gene, probe type (fluorophore) and excitation/detection wavelengths were as follows: *MgPa* (MG), PlexProbe 1 (FAM), 465–510 nm; *GCporA* (GC), linear PlexZyme Probe 1 (FAM), 465–510 nm; *CTcry* (CT), PlexProbe 2 (JOE), 533–580 nm; *GCopa* (GC), linear PlexZyme Probe 2 (JOE), 533–580 nm; *TVK* (TV), PlexProbe 3 (Atto Rho101), 533–610 nm; and IC, linear PlexZyme Probe 3 (Atto Rho101), 533–610 nm. Data generated using the linear PlexZyme probes may be either quantitative (in real-time) or qualitative (endpoint), whereas data generated using the PlexProbes is qualitative (endpoint). Cross-reactivity of all primers, partzyme and probe sequences were first analyzed in silico using BLAST to ensure specificity. The target sequences are listed in S2 Table.

## Results

### PlexProbe PCR method and model assay detecting CT/GC in a single channel

**Fig 1:**
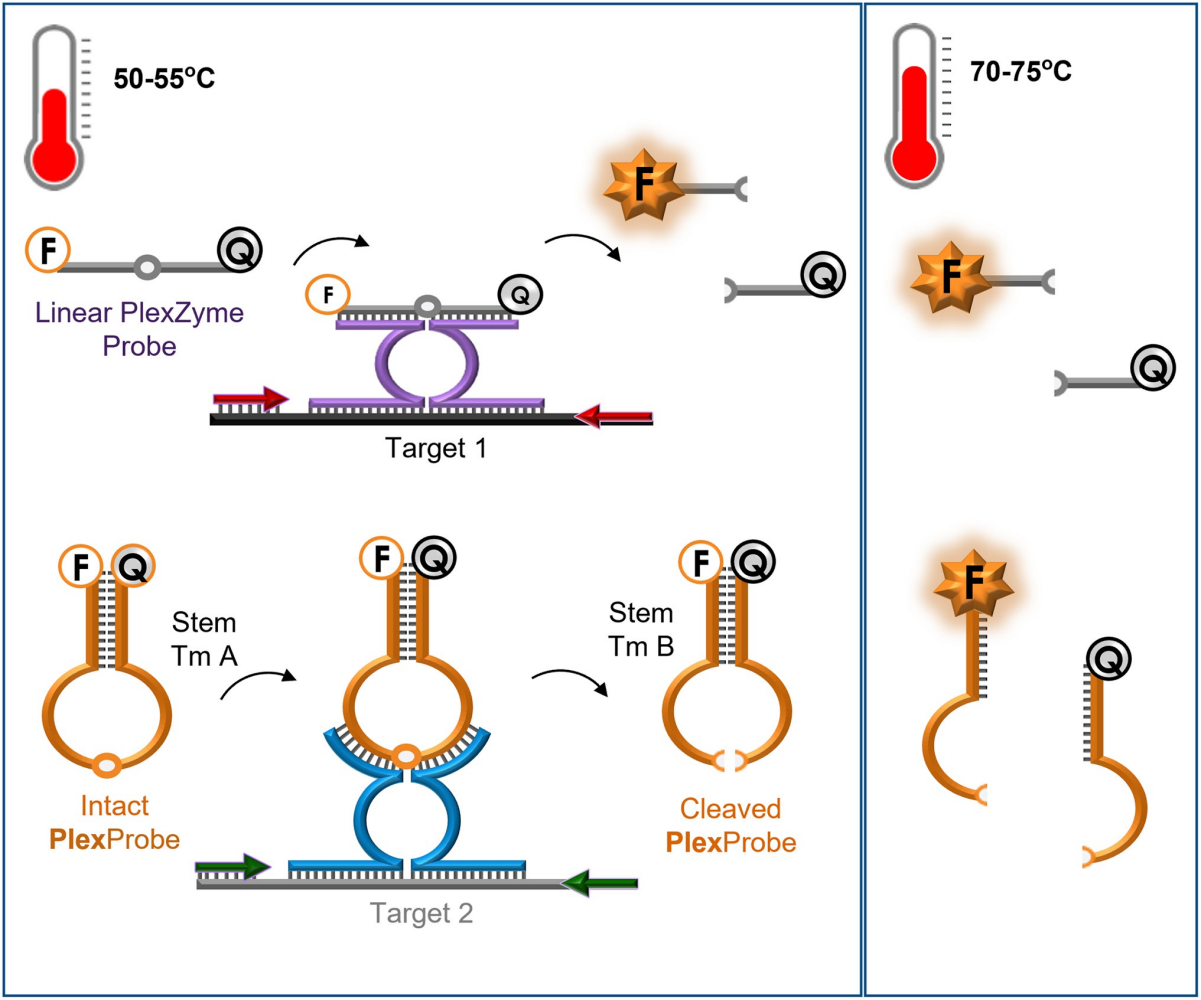
Overview of PlexProbe PCR method. Structure of Linear PlexZyme Probes (grey) and PlexProbes (orange) prior to, and following, cleavage by PlexZymes. Left Hand Side: Structures at temperature range 1 (50–55°C). Right Hand Side: Structures at temperature range 2 (70–75°C). Linear PlexZyme Probes and PlexProbes are labelled with the same fluorophore (F) and quencher (Q) dye pair. During PCR, PlexZyme 1 (purple) assembles in the presence of Target 1 (black) and PlexZyme 2 (blue) assembles in the presence of Target 2 (grey). Intact PlexProbes contain a stem region with a Tm A whereas Cleaved PlexProbes have stems with a Tm B which is lower than Tm A.

The novel PCR method used in this study combines PlexProbes with standard linear PlexZyme probes for the detection and differentiation of two targets in a single fluorescent channel. Both of these probe types are universal reporter probes that do not bind to the target and instead bind to intermediate target-dependant catalytic nucleic acids known as PlexZymes [7]. The features of these probes and the strategy for fluorescent signal generation is illustrated in Fig 1.

The strategy uses two types of probes, namely Linear PlexZyme Probes and PlexProbes. Both probe types are labelled with the same fluorophore and quencher dye pair, and each contains a unique, universal nucleic acid substrate which is cleavable by a specific PlexZyme. In a single PCR mix, PlexZyme 1 can assemble from its component partzymes in the presence of Target 1 and cleave multiple linear PlexZyme Probes and PlexZyme 2 can assemble from its component partzymes in the presence of Target 2 and cleave multiple PlexProbes. Cleavage of linear PlexZyme Probes separates the fluorophore and quencher causing an increase in fluorescence that can be detected across a broad range of temperatures during PCR (50–75°C).

PlexProbes are an extension of linear PlexZyme probes wherein the substrate is attached to a stemmed region of complementary base pairs forming a stem-loop structure. The length and composition of the stemmed region controls the Tm which in turn determines the temperature where each PlexProbe will fluoresce or remain quenched. This design allows the fluorescence intensity to be regulated in response to the presence of target at defined temperatures. Intact PlexProbes contain a stem region with a Tm A and cleavage of the loop results in Cleaved PlexProbes that have stems with a Tm B which is lower than Tm A. During PCR, the stem of both Intact and Cleaved PlexProbes remain hybridized and quenched at a first temperature typically in the range of 50–55°C. At a second temperature, for example between 70 to 75°C, the stem of Intact PlexProbes remain hybridised and quenched, whereas the stems of Cleaved PlexProbes separate and generate fluorescence indicative of the presence of the target. In this manner, PlexProbes are both target and temperature dependent with respect to their capacity to fluoresce. Overall, if the fluorescence increases during PCR at the first temperature this indicates the presence of target 1 and if the increase in fluorescence observed at temperature 2 is greater than at temperature 1 this indicates the presence of target 2. Data generated using the linear PlexZyme probes may be either quantitative (in real-time) or qualitative (endpoint), whereas data generated using the PlexProbes is qualitative (endpoint).

To demonstrate this strategy, we developed a model assay detecting CT and GC in a single channel. Both the PlexProbe and the linear probe were labelled with the same fluorophore (JOE) and, following cleavage by their specific PlexZymes, are detectable in the same qPCR channel. The presence of Target 1 (CT) and/or Target 2 (GC) in a sample are differentiated based on fluorescent signal intensity at two different temperatures where CT is detected using a linear PlexZyme probe at Temperature 1 (52°C) and GC is detected using a PlexProbe at Temperature 2 (74°C). In terms of PCR amplification and detection, the method is the same as the standard PlexPCR approach, however, one of the linear probes is replaced with a PlexProbe and an additional fluorescence measurement was acquired at a second temperature prior to and upon completion of PCR.

**Fig 2:**
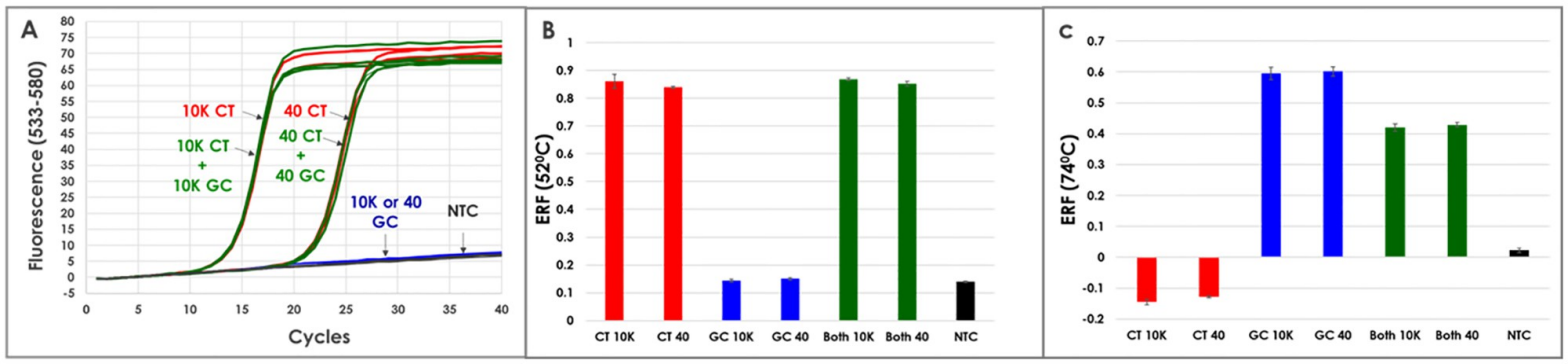
Results of model assay detecting CT/GC in a single channel. The protocol combined one linear probe reporting on CT together with one PlexProbe reporting on GC where both probes were labelled with the same fluorophore (JOE). Triplicate reactions contained either 10,000 (10K) or 40 copies of CT template (red), 10,000 or 40 copies of GC template (blue), 10,000 or 40 copies each of both CT and GC (green) or no template (NTC) (black). (A) PCR amplification curves acquired at 52°C demonstrate real-time quantitative results for CT in the presence or absence of GC. (B) Endpoint relative fluorescence (ERF) obtained post-PCR at 52°C, calculated according to Formula A, allows qualitative detection of CT in samples in the presence or absence of GC. (C) ERF obtained post-PCR at 74°C, calculated according to Formula B, allows qualitative detection of GC in samples in the presence or absence of CT. Error bars represent the standard deviation.

The results in Fig 2 show the presence of CT and/or GC in a sample were easily differentiated based on fluorescent signal intensity at two different temperatures where CT was detected using a linear PlexZyme probe at 52°C and GC was detected using a PlexProbe at 74°C. Data in Fig 2A shows identical Cq values for given numbers of copies of CT regardless of the presence or absence of GC in the sample. As such, the presence of additional “off target” amplicons do not result in a shift in Cq for the target of interest. Importantly, no amplification curves were generated for reactions lacking CT. The data shown in Fig 2B demonstrates that qualitative endpoint analysis is also possible; and, similarly to the real -time analysis, as little as 40 copies of the CT could be distinguished regardless of the presence or absence of GC.

The post-PCR differential fluorescence acquired at 74°C and 52°C allowed for qualitative determination of the presence or absence of GC in a sample (Fig 2B). Since cleaved linear probes fluoresce at all temperatures, their contribution must be subtracted from the total fluorescence at 74°C. The Formula B described in the methods section was used to determine fluorescence associated with cleaved PlexProbes only. The results in Fig 2C show the endpoint relative fluorescence for samples containing CT only, GC only or both CT and GC in varied concentrations.

### Analysis of clinical specimens using the six-plex PP-STI assay

The PP-STI assay was evaluated for detection of CT, GC, TV and MG from DNA extracted from 441 clinical specimens and compared to a commercially available reference test (AllPlex^™^ Essential STI (Seegene)). The PP-STI assay included an IC which amplified efficiently in 337 extracts indicating these lacked inhibitors and were amenable to amplification. Four extracts did not amply the IC and were excluded from further analysis. Positive results were analysed according to the four specific pathogens with co-infections considered as two results. In total, 337 samples and 1348 results were statistically evaluated.

Dual gene or confirmatory testing is recommended for GC diagnosis for extragenital specimens and in low prevalence populations [22–24]. Since the GC genome only contains a single copy of the *porA* pseudogene compared to around 11 copies of the *opa* loci [25], specimens in this study were deemed positive for GC if both the *opa* and the *porA*, or only the *opa* gene was detected. In total, four GC positive samples were positive for the *opa* gene but negative for *porA* gene.

Analysis of the clinical specimens using the PP-STI assay resulted in an overall agreement with the comparator of 99.5% (1342/1348) (Table 3**)** with high positive percentage agreement (PPA) and negative percentage agreement (NPA) for all targets.

**Table 3:** Analysis of 337 clinical specimen using the PP-STI assay and the AllPlex^™^ STI essential assay.

	PP-STI assay		AllPlex^™^ STI essential assay	PPA	NPA	Agreement	Kappa
	Positive	Negative					
**MG**	69	3	**Positive** 72	97.9%	99.4%	99.1%	97.3%
	0	265	**Negative** 265				
**GC**	78	0	**Positive** 78	99.4%	99.8%	99.7%	99.2%
	1	258	**Negative** 259				
**TV**	43	0	**Positive** 43	100%	100%	100%	100%
	0	294	**Negative** 294				
**CT**	49	2	**Positive** 51	98.0%	99.7%	99.4%	97.7%
	0	286	**Negative** 286				

Amongst discrepant samples, five were negative by the PP-STI assay, of which two were positive for CT and three were positive for MG according to the comparator. A further sample indicated positivity for both TV and GC using the PP-STI assay whereas the comparator detected TV only. Whilst there was insufficient material to retest three negative samples (one CT and two MG positive), re-testing of the remaining discrepant samples (one CT and one MG positive) using the PP-STI assay resulted in concordant results between the two tests. These discordant samples had late Cq values using the comparator test (Cq >35.5) and the nucleic acid extracts had been stored for up to two years.

## Discussion

In the present study, we showcase a universal PCR probe technology using a model CT/GC duplex assay and a prototype STI six-plex panel. Both assays provided simultaneous detection and differentiation of two targets in each fluorescent channel. These data demonstrate a strategy whereby more targets can be combined in a single diagnostic test, essentially doubling the multiplexing capacity of PCR instruments. This is particularly valuable for syndromic based testing such as STIs, where detection of multiple pathogens with overlapping symptoms, and where highly multiplexed panels could expedite diagnosis. The strategy allowed for the quantification and qualitative analysis of one target, and qualitative detection of a second target, in each channel without the need for time-consuming MCA.

Using the CT/GC duplex model assay, we demonstrated the PlexProbe chemistry to be highly sensitive, easily detecting 40 copies of either target. When samples containing both CT and GC were monitored in real-time, results showed that the quantification of CT was unaffected by the presence of GC as evidenced by no shift in the threshold cycle number. This highlights how fluorescence from PlexProbes can be easily controlled in both a target and temperature dependent manner. Further, we demonstrated that the addition of GC did not impact results when endpoint relative fluorescence was used to detect CT in a sample or vice versa.

The PlexProbe method described in this study provides a format allowing quantification of some targets and qualitative detection of others without increasing the overall time to result. There is also the option to perform endpoint analysis for both targets measured in a single channel which creates the potential for PCR formats using very rapid cycling with fluorescence only acquired prior to, and following, amplification. This shorter turnaround time is paramount for medical diagnostics as it enables faster diagnosis and the ability for testing laboratories to process more samples each day.

The ability of PlexProbes to facilitate multiplex qPCR detection was further demonstrated using a prototype assay that detected six independent targets in only three fluorescent channels. Two targets were identified and distinguished in each channel, with one target detected in real-time and the other at endpoint. In the initial clinical evaluation of the PP-STI reported here, a high level of concordance was observed between results obtained with the PP-STI assay and a commercially available comparator, indicating that the PlexProbe technology is sufficiently robust for clinical applications.

The three PlexProbes and three linear PlexZyme probes used in the prototype STI assay were all universal in nature (i.e. target independent) and hence can be cleaved by any target specific PlexZyme. As such, this panel of probes could theoretically be used for any six targets in PCR assays detecting multiple targets per fluorescent channel; and since these universal components can be manufactured in bulk, this could lead to cost savings if used across a range of assays. Further, since detection can be consolidated into only three channels, assays of this type could be used on instruments with limited numbers of independent fluorescence channels, such as compact and/or POC amplification systems.

## Conclusion

PlexProbes provide a new tool for increasing the capacity to multiplex on existing PCR platforms without compromising sensitivity or specificity. These universal probes function independent of target and can be used to build PCR panels for detecting any nucleic acid targets of choice without modification. Their capacity to function under common cycling conditions could expedite new test development. Further, since more targets can be analysed per channel, these probes can be used on a broader range of instruments including POC devices that have a limited number of detection channels. By detecting more targets in a single test, this approach has the potential to conserve time, labour, sample volume and reagent cost.

## Supporting information

S1 Table Oligonucleotide sequences used in the model GC/CT duplex. (DOCX)

S2 Table Target sequences used in the PP-STI assay. (DOCX)
