# Supplementary material for: PlexProbes enhance qPCR multiplexing by discriminating multiple targets in each fluorescent channel
Source: PLoS One. 2022 Mar 9;17(3):e0263329. doi: 10.1371/journal.pone.0263329 (PMC8906580; doi:10.1371/journal.pone.0263329)
Supplement: S1 Table — (DOCX) [file pone.0263329.s001.docx]

**Supporting Information**

**Table S1** Oligonucleotide sequences used in the model GC/CT duplex PlexProbe test where upper case denotes DNA, Lowercase denote RNA bases, P denotes 3’ phosphorylation. Both the probes were labelled with JOE fluorophore at the 5` end and Iowa Black® FQ at the 3` end.

| **Oligonucleotide** | **Sequence (5’-3’)** |
| --- | --- |
| 5’ CTcry primer | AATATCATCTTTGCGGTTGCGTGTCC |
| 3’ CTcry primer | GCTGTGACGGAGTACAAACGCC |
| CTcry Partzyme A | TCCTGTGACCTTCATTATGTCGACAACGAGAGGAAACCTT-P |
| CTcry Partzyme B | TGCCCAGGGAGGCTAGCTGAGTCTGAGCACCCTAGGC-P |
| Linear Probe | AAGGTTTCCTCguCCCTGGGCA |
| 5 GCopa primer | GTGTTGAAACACCGCCCGG |
| 3 GCopa primer | GCTCCTTATTCGGTTTGACCGG |
| NGopa Partzyme A | CCGGAACCCGATATAATCCGCACAACGAGAGGGTCGAG |
| NGopa Partzyme B | GGACGAGGGAGGCTAGCTCCTTCAACATCAGTGAAAATCTTTT |
| PlexProbe | AACGACAATGGCCTTTTCTCGACCCTCguCCCTCGTCCTTTTGGCCATTGTCGTT |

|  |  |  |  |  |  |
| --- | --- | --- | --- | --- | --- |
