# Supplementary material for: PlexProbes enhance qPCR multiplexing by discriminating multiple targets in each fluorescent channel
Source: PLoS One. 2022 Mar 9;17(3):e0263329. doi: 10.1371/journal.pone.0263329 (PMC8906580; doi:10.1371/journal.pone.0263329)
Supplement: S2 Table — (DOCX) [file pone.0263329.s002.docx]

**Supporting Information**

|  |  |  |  |  |  |
| --- | --- | --- | --- | --- | --- |

**Table S2** Target Sequences used in the PP-STI assay

| **Target** | **Genbank Accession No** | **Sequence (5’-3’)** |
| --- | --- | --- |
| CT | HE603218.1 | AATATCATCTTTGCGGTTGCGTGTCCCGTGACCTTCATTATGTCGGAGTCTGAGCACCCTAGGCGTTTGTACTCCGTCACAGC |
| GC opa | AE004969.1 | TATTGTGTTGAAACACCGCCCGGAACCCGATATAATCCGCCCTTCAACATCAGTGAAAATCTTTTTTTAACCGGTTAAACCGAATAAGGAGC |
| GC porA | AJ010733.1 | GCAGCATTCAATTTGTTCCGAGTCAAAACAGCAAGTCCGCCTATACGCCTGCTACTTTCACGCTGGAAAGTAATCAGATGAAACCAGTTCCG |
| TV | XM_001297477 | GTTTGTGTCTCGTGCCATAGTCGAGCAAAATACAGACAAACAGGTTATATATGAGTTTGAGACCAAGAATGGTGTAACTCGACCTGTCCGATTCAAAGACTCCGAGGGCGACCATGAAAT |
| MG | GU226203.1 | AATACCTTGATGGTCAGCAAAACTTTGCAATCAGAAGGTATGATAACAACGGTAGAGCTTTATATGATATTAACTTAGCAAAAATGGAAAACCCCTCAACGGTGCAAAGGGGTTTAAATGGCGAGCC |
